# *R*-Phycoerythrin Induces SGC-7901 Apoptosis by Arresting Cell Cycle at S Phase

**DOI:** 10.3390/md14090166

**Published:** 2016-09-12

**Authors:** Huixin Tan, Shiyong Gao, Yan Zhuang, Yanhong Dong, Wenhui Guan, Kun Zhang, Jian Xu, Jingru Cui

**Affiliations:** 1 Department of Pharmacy, The Fourth Affiliated Hospital of Harbin Medical University, Harbin 150001, China; thxydsy@163.com (H.T.); dyhhrb@163.com (Y.D.); guanwxydsy@163.com (W.G.); zhangkunydsy@163.com (K.Z.); xujianydsy@163.com (J.X.); 2 The Institute of Materia Medica, The Research Center of Life Sciences and Environmental Sciences, Harbin University of Commerce, Harbin 150076, China; zzyilai@163.com

**Keywords:** *R*-phycoerythrin, apoptosis, cell cycle arrest, human gastric cancer, cell cycle-related protein

## Abstract

*R*-Phycoerythrin (*R*-PE), one of the chemical constituents of red algae, could produce singlet oxygen upon excitation with the appropriate radiation and possibly be used in photodynamic therapy (PDT) for cancer. Documents reported that *R*-PE could inhibit cell proliferation in HepG2 and A549 cells, which was significative for cancer therapy. This is due to the fact that *R*-PE could kill cancer cells directly as well as by PDT. However, little is known about the cytotoxicity of *R*-PE to the SGC-7901 cell. In this study, it has been found that *R*-PE could inhibit SGC-7901 proliferation and induce cell apoptosis, which was achieved by arresting the SGC-7901 cell at S phase. CyclinA, CDK2 and CDC25A are proteins associated with the S phase, and it was found that *R*-PE could increase the expression of cyclin A protein and decrease the expression of CDK2 and CDC25A proteins. Thus, it was concluded that *R*-PE reduced the CDK2 protein activated through decreasing the CDC25A factor, which reduced the formation of Cyclin-CDK complex. The reduction of Cyclin-CDK complex made the SGC-7901 cells arrest at the S phase. Therefore, *R*-PE induced apoptosis by arresting the SGC-7901 cell at S phase was successful, which was achieved by the expression of the CDC25A protein, which reduced the CDK2 protein actived and the formation of Cyclin-CDK complex.

## 1. Introduction

**Figure 1:**
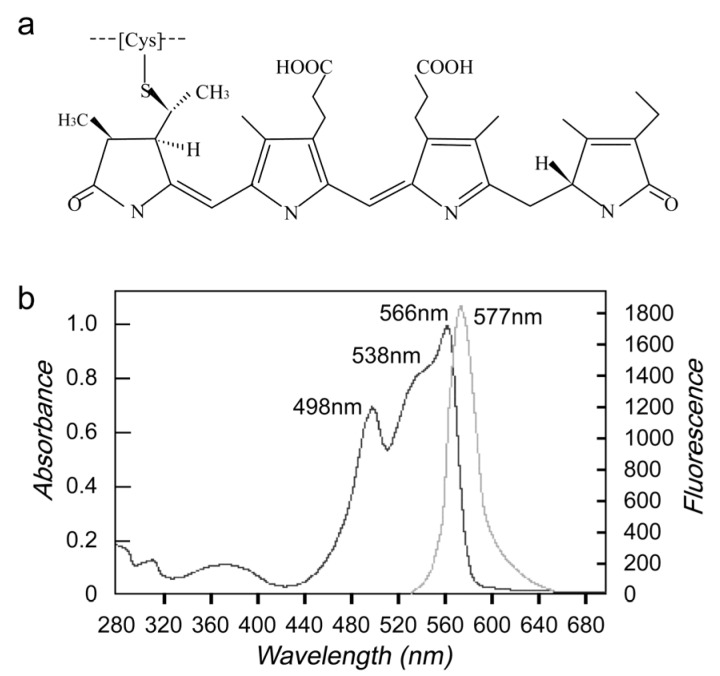
Struction of *R*-phycoerythrin (*R*-PE) (**a**) and absorption maximum (**b**).

Phycoerythrin (PE) is one of four classes of phycobili protein (phycoerythrin, phycocyanin, phycoerythrocyanin and allophycocyanin) [1], which are divided into four main classes on the basis of their absorption spectra, *R*-phycoerythrin (*R*-PE, peaks at 499, 565 nm and a shoulder peak at 545 nm), C-phycoerythrin (C-PE, peak at 565 nm), B-phycoerythrin (B-PE, peaks at 545 nm, 565 nm and a shoulder at 499 nm), and CU-phycoerythrin (CU-PE, peaks at about 498 nm, 540 nm and/or 565 nm) [1,2,3,4]. *R*-phycoerythrin (*R*-PE), molecular weight 240,000 daltons, is a phycobiliprotein isolated from proprietary red algae. *R*-PE is composed of a carrier protein connecting open-chain tetrapyrrole compound with thioetherbond. The protein has an (alpha-beta)_6_ gamma composition. Both alpha- and beta-subunits are approximately 20,000 daltons, and the gamma-subunits approximately 30,000 daltons (Figure 1a). The purified *R*-PE showed three absorption peaks at 498 nm, 538 nm (shoulder peak), 566 nm and one fluorescent emission maximum at 577 nm (Figure 1b) [5].

*R*-PE is commonly used as a fluorescent label probe [6,7,8,9], as biological and clinical assays [10] and for in-flow cytometry [8,11]. It is also used as a pigment or natural food dye [12]. *R*-PE can produce singlet oxygen upon excitation with appropriate radiation and hence could be useful in photodynamic therapy (PDT) for cancer [13]. Literature states numerous biological activities of *R*-PE, such as antioxidant activity [14], anti-cancer properties [15], anti-carcinogenic nature [13,16], and anti-aging activity [17] et al. It was observed that it also shows proliferation inhibition on the SGC-7901 cell by arresting cell cycle phase, which was not reported by other research.

## 2. Results

### 2.1. Effect of R-PE on SGC-7901 Cell Viability

To confirm the inhibitory effect of *R*-PE on SGC-7901 human gastric cancer cell growth, cell growth was measured using an 3-(4,5-dimethylthiazol-2-yl)-2,5-diphenyltetrazolium bromide (MTT) assay. As showed in Table 1, *R*-PE proved that it could inhibit the cell proliferation in SGC-7901 human gastric cancer cells, and the IC_50_ value of cell growth inhibition was 2.55 μM.

### 2.2. Effect of R-PE on SGC-7901 Cell Morphology and Cell Nuclei Morphology

**Figure 2:**
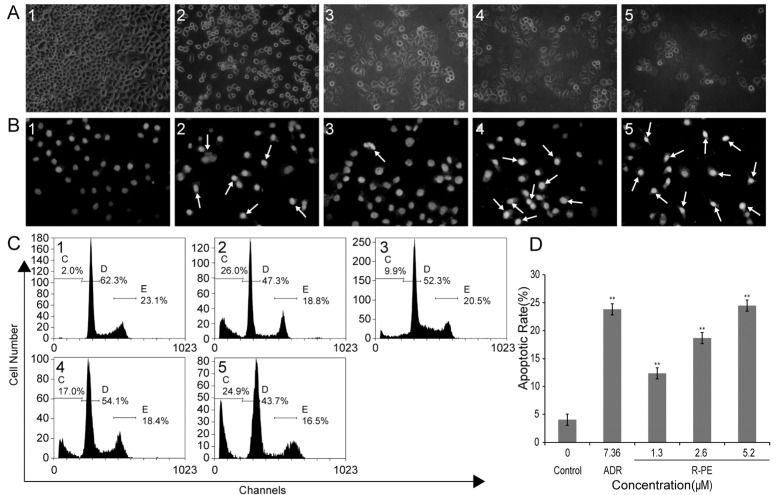
*R*-PE induces apoptosis in SGC-7901 cells. SGC-7901 cells were treated with 1.3, 2.6, and 5.2 μM *R*-PE for 48 h. (**A**) morphological change of SGC-7901 cells observed under an inverted light microscope (200×); (**B**) nucleolus morphologic changes observed by fluorescent microscope (200×). Apoptotic cells are observed, the nuclei exhibited bright-condensed chromatin, which was showed by arrow; (**C**) apoptotic rate measured by propidium iodide (PI) staining and flow cytometry assay; and (**D**) the histogram of apoptotic rates of SGC-7901 cells induced by *R*-PE. ** *p* < 0.01 vs. control. (**1**) control; (**2**) 7.36 μM adriamycin (ADR); (**3**) 1.3 μM *R*-PE; (**4**) 2.6 μM *R*-PE; and (**5**) 5.2 μM *R*-PE.

An inverted light microscope was adopted to observe the effect that *R*-PE had on the cellular morphology. The SGC-7901 cells of the control group grew well, and the skeletons were clear, with most of the cells being polygonous (Figure 2(A1)). Cells treated with *R*-PE showed morphological changes of apoptosis, such as detachment from substratum and cell rounding, cell shrinkage and decrease in cell number (Figure 2(A3–5)).

The morphology of the nuclei was observed under a fluorescent microscope. The dye used was Hoechst 33258 and was evenly distributed in the cells of control group, which indicated that the chromatin was equally distributed in the nucleus. The cells treated with *R*-PE presented the morphological features of apoptotic cells, such as emitting brighter fluorescence (nuclear condensation identified by propidium iodide (PI) staining) [18,19] and nuclear membrane cracking (the nucleus dispersion) (Figure 2(B3–5)).

### 2.3. R-PE Induces Apoptosis Rate of SGC-7901 Cells

To quantify the apoptosis rate of *R*-PE on SGC-7901 cells, the PI staining and flow cytometry assay was employed. The data showed that *R*-PE induced cell apoptosis of SGC-7901 cells in a dose-dependent manner (Figure 2C,D and Table 2). When the cells were treated with 1.3, 2.6, 5.2 μM *R*-PE for 48 h, the average proportion of sub-G_0_ phase cells (apoptosis cells) significantly increased from 4.03% ± 2.64% in control to 12.37% ± 4.62%, 18.67% ± 3.06%, 24.50% ± 1.64%, respectively (Table 2, Figure 2C). Figure 2D shows the graphic representation of the increase in the apoptosis rate with an increase in the dose of *R*-PE.

### 2.4. Effect of R-PE on the Cell Cycle in SGC-7901 Cells

**Figure 3:**
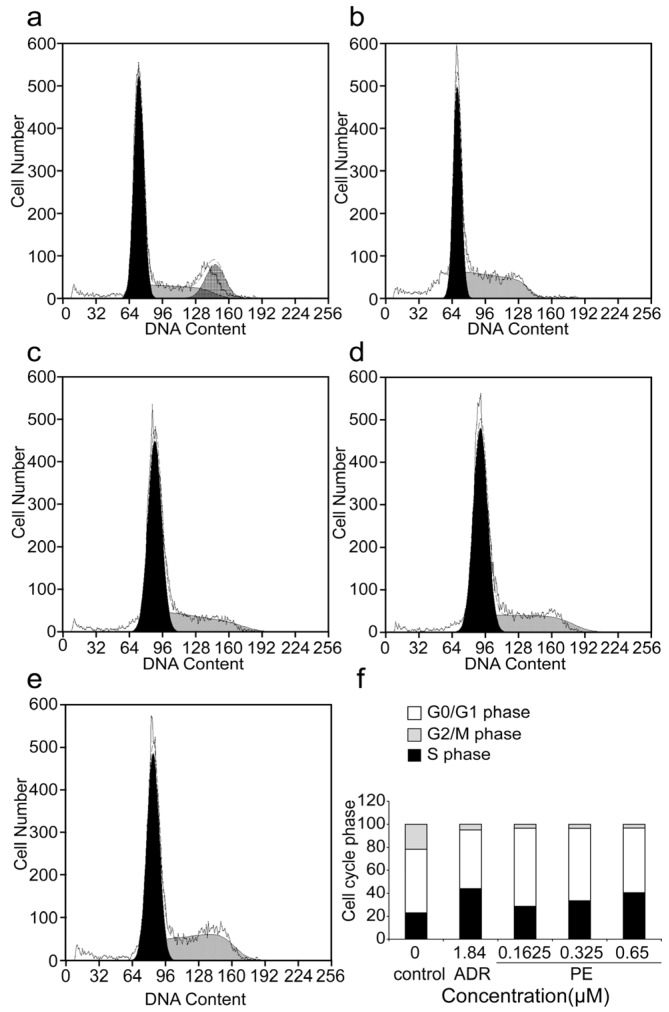
The effects of *R*-PE on cell cycle arrest of SGC-7901 cells. (**a**) control; (**b**) 1.84 μM ADR; (**c**) 0.1625 μM *R*-PE; (**d**) 0.325 μM *R*-PE; (**e**) 0.65 μM *R*-PE; and (**f**) histogram. PI staining and flow cytometer assay were adopted to measured the cell cycle of SGC-7901 cell treated by 0.1625, 0.325, 0.65 μM *R*-PE for 24 h. *R*-PE could arrest SGC-7901 cells at S phase in a dose-dependent manner.

Analysis of the cell cycle arrest in SGC-7901 cells was carried out using a flow cytometer assay (Figure 3). The results showed that the number of cells in the S phase significantly increased from 23.17% ± 3.50% in the control group to 28.60% ± 1.66%, 33.43% ± 2.95% and 40.50% ± 2.66% in the *R*-PE treated groups (showed in Table 3), The Figure 3f showed the graphic representation of the increase of the S phase. The population of SGC-7901 cells at the G_2_/M phase was significantly reduced after *R*-PE treatment, which concurred that the *R*-PE could arrest the SGC-7901 cells at the S phase.

### 2.5. Effect of R-PE on Cycle-Associated Protein Expression in SGC-7901 Cells 

**Figure 4:**
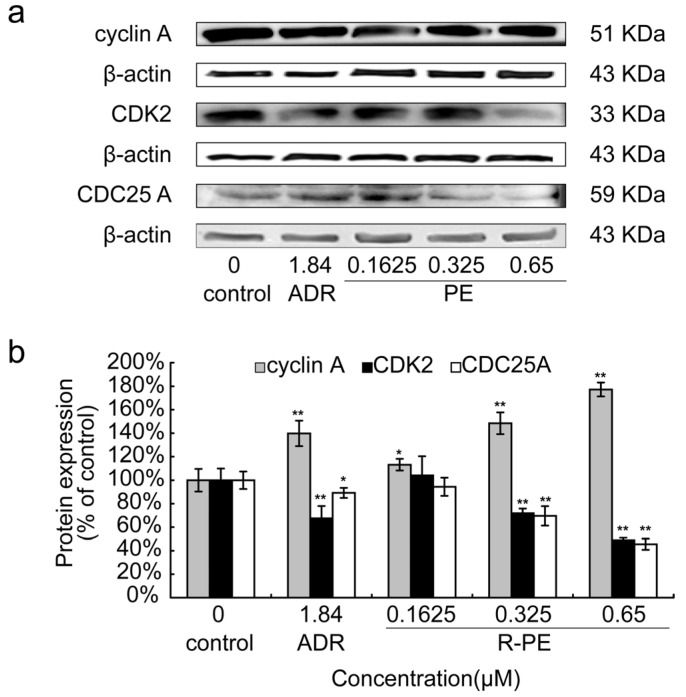
Effect of *R*-PE on the expression level of S phase cycle-related protein in SGC-7901 cells. SGC-7901 cells were treated with 0.1625 μM, 0.325 μM, 0.65 μM of *R*-PE for 24 h and S phase apoptosis-associated proteins were examined by Western blot assay as described in Materials and Methods. (**a**) electrophoretogram of Western blot and (**b**) histogram shows that *R*-PE could increased the protein expression level of cyclin A and decreased the protein level of CDK2 and CDC25A in a dose-dependent manner. * *p* < 0.05, ** *p* < 0.01 vs. control.

To further confirm the cell cycle arrest mediated by *R*-PE in SGC-7901 cancer cells, S phase cycle-related proteins (including cyclinA, CDK2, CDC25A) were examined. As shown in Figure 4a, *R*-PE significantly increased the protein expression of cyclin A and decreased the expression of CDK2 and CDC25A protein. The data in Figure 4b showed that *R*-PE affected the cyclin A, CDK2 and CDC25A protein expression in a dose-dependent manner.

## 3. Discussion

Red algae is one of the oldest groups of eukaryotic algae [20] and also one of the largest phyla of algae, with more than 5000–6000 distinct species [21]. They are almost exclusively multicellular marine algae. Most red algae, such as dulse and laver, are also used as food throughout the world, especially in Asia. The red algae is also used to make other products such as agar [22], carrageenans [23] et al. Red algae get their color from a specific pigment called phycoerythrin. This pigment absorbs blue light and reflects red, hence the color. The *R*-PE studied in this paper was prepared from red algae.

Literature reported that the red alga can inhibit cell proliferation of cancer cell [24,25,26]. The antitumor activity of red alga is related to various chemical constituents of red alga such as kappa-Carrageenan [27], polysaccharide [28], Sterol Fraction [29], sterol glycosides [30] and phycoerythrin. 

Phycoerythrin is divided into four main classes on the basis of their absorption spectra: *R*-phycoerythrin (*R*-PE), C-phycoerythrin (C-PE), B-phycoerythrin (B-PE) and CU-phycoerythrin (CU-PE). CU-PE has similar chromophores and absorption properties as *R*-PE and used to be classified as *R*-PE. Literature states that C-PE and *R*-PE can inhibit cancer cell proliferation but none of B-PE. C-PE purified from marine *Lyngbya* sp. induces apoptosis in A549 human lung carcinoma cells. *R*-PE is the chemical constituent of red algaae and has been used to treat many types of cancer by Photodynamic Therapy (PDT). The *R*-PE subunit can easily accumulate in tumor cells because the tumor cells proliferate faster than normal tissue cells, which can be activated after 496 nm light irradiation. It can then destroy the tumor cell by photochemical reaction and can be applied in the treatment of liver cancer [13] and cervical cancer [16]. PDT using *R*-PE was proved to be effective in animal experiments [13,16]. In vitro experimentation showed that *R*-PE could induce the apoptosis in HepG2 cells and A549 cells by arresting the cell at G_2_/M phase [15]. The results of this study showed that *R*-PE could inhibit SGC-7901 cell proliferation and the IC_50_ value recorded was 2.55 μM (Table 1). The findings regarding the proliferation inhibition of *R*-PE are very significant for the treatment of cancer because *R*-PE is mainly used for PDT at present, and if the *R*-PE can kill cancer cell as well as be used in PDT, it has the effect of icing on the cake for cancer treatment.

It was also found that *R*-PE could destroy the morphology of cell and cell nucleus (Figure 2A,B). This showed *R*-PE may induce cell apoptosis. PI stain and flow cytometer assay were adopted to determine the apoptosis rate of *R*-PE in SGC-7901 cells, and the results showed that the apoptosis rate increased from 4.03% ± 2.64% in control to 12.37% ± 4.62%, 18.67% ± 3.06%, 24.50% ± 1.64% in 1.30 μM, 2.60 μM, and 5.20 μM *R*-PE group (Table 2, Figure 2C,D). This showed that *R*-PE could induce the apoptosis of SGC-7901 cells.

Cell cycle arrest is an important cause of cell apoptosis, Senthilkumar reported that *R*-PE could induce cell cycle arrest in HepG2 and A549 cells [15]. The effect of *R*-PE on cell cycle was observed, and it was found that *R*-PE arrested SGC-7901 cells at the S phase (Table 3, Figure 3**)**, this is different from that of *R*-PE, which arrested the HepG2 and A549 cells at G2/M phase [15]. Therefore, the arresting effect was different in various types of cancer cell lines.

CDK and Cyclin are the core factors of endogenous regulation and control of cell cycle. CyclinA, CDK2 and CDC25A are cell factors associated with the S phase of cells. CDC25A could activate the CDK2, which in turn activates the Cyclin-CDK complex. This could promote the operation of the cell cycle and therefore high expression of CDC25A protein could cause rapid cell growth [31,32].

The results of this study showed that the *R*-PE can decrease the expression of CDC25A and CDK2 protein, and increase the expression of CyclinA (Figure 4). It was deduced that *R*-PE reduced the CDK2 protein activated through decreasing the CDC25A factor, which reduced the formation of Cyclin-CDK complex. The reduction of Cyclin-CDK complex made the SGC-7901 cells arrest at the S phase. Simultaneously, the expression of free Cyclin A protein would increase because of the reduction of Cyclin-CDK complex, which was confirmed in the experiments (showed in Figure 4).

Therefore, *R*-PE induced apoptosis by arresting the SGC-7901 cell at S phase was achieved by decreasing the expression of the CDC25A protein, reducing the CDK2 protein activated and the formation of Cyclin-CDK complex.

## 4. Materials and Methods

### 4.1. Chemicals and Other Reagents

Phycoerythrin (*R*-PE, A_max_/A_280nm_ > 5.0) was purchased from Hongrui Biotech Company (Pan’an County, Zhejiang, China). MTT, trypsin, PI, RNase A, Tween-20, glycine, acrylamide, methylene diacrylamide and Tris were purchased from the Sigma-Aldrich (St. Louis, MO, USA). RPMI-1640 cell culture medium and trypsin were purchased from Gibco (Grand Island, NY, USA). Doxorubicin hydrochloride was purchased from Pfizer Pharmaceuticals Limited (Brooklyn, NY, USA). Fetal calf serum (FCS) was purchased from Thermo Fisher Scientific (Waltham, MA, USA). Hoechst33258, penicillin-streptomycin, cell lysis buffer, and tetramethylethylenediamine (TEMED) were obtained from the Beyotime Institute of Biotechnology (Shanghai, China). DAB Horseradish Peroxidase Color Development Kit was purchased from ZSGB-BIO (Beijing, China). All other chemicals and solvents used were the highest purity grade.

### 4.2. Cell Line and Culture Conditions

The human gastric carcinoma SGC-7901 cell line was obtained from the Institute of Cancer Prevention and Control of Harbin Medical University (Harbin, China). The cells were grown in RPMI-1640 medium containing 10% fetal bovine serum at 37 °C in a humidified atmosphere containing 5% CO_2_. Cells were harvested by trypsinization with 0.25 mg/mL trypsin solution and suspended in culture medium before use.

### 4.3. Cell Viability and Cytotoxicity 

Exponentially growing cells were harvested using 0.25% (*w*/*v*) trypsin and dispensed into 96 well plates at 1.0 × 10^4^ cells/well. After 24 h incubation, the cells were exposed to different concentrations of *R*-PE for 72 h. Then, the medium was discarded, 100 μL of MTT solution (1 mg/mL) was added and incubated for 4 h, and dimethyl sulfoxide (DMSO) was used to solubilize the water-insoluble purple formazan crystals. Then, the absorbance of the solution was read at a wavelength of 570 nm [33] using a microplate reader (Bio-Rad, Fitchburg, WI, USA). The percentage inhibition was calculated by comparing optical density (OD) value of drug-treated cells with that of untreated cells. All experiments were repeated six times in six wells of the microplate. The half maximal inhibitory concentration (IC_50_) was calculated by the Logit method [34,35].

### 4.4. Cell Morphology and Cell Nuclear Morphology Observation (Hoechst 33258)

Cells were seeded in 6-well plates and after 24 h incubation, the medium was removed and replaced with a medium containing *R*-PE (1.3 μM, 2.6 μM, 5.2 μM) for 48 h. The cell morphology was observed by taking photos under an inverted microscope equipped with a digital camera. The cells were then fixed with ethanol/acetic acid (3:1) at 4 °C for 20 min, and stained with Hoechst 33,258 for 30 min in the dark. The nuclear morphology was observed under a fluorescence microscopy (Leica, Wetzlar, Germany).

### 4.5. Flow Cytometric Analysis of Apoptosis

Cells were seeded in 6-well plates, and incubated for 24 h, The medium was then removed and replaced with a medium containing the *R*-PE (1.3, 2.6, 5.2 μM) for 48 h. Cells were harvested and washed with phosphate buffer saline (PBS) twice, and then fixed in 1 mL cold 70% ethanol at 4 °C for 12 h. Fixed cells were washed with PBS and re-suspended in 1 mL PI staining solution (1 mg/mL sodium citrate, 50 μg/mL PI, 10 μg/mL RNase A, 0.5% Triton X-100). The cells were then incubated at 37 °C for 30 min in the dark. The data acquisition and analysis of the cell apoptotic rate was performed using MultiCycle software of flow cytometry (Beckman Coulter, XL, Indianapolis, IN, USA).

### 4.6. Flow Cytometric Analysis of Cell Cycle

Cells were seeded in 6-well plates, and after 24 h incubation, the medium was removed and replaced with a medium containing the *R*-PE (0.1625 μM, 0.325 μM, 0.65 μM) for 48 h. Cells were harvested and washed with PBS twice, and then fixed in 1 mL cold 70% ethanol at 4 °C for 12 h. Fixed cells were washed with PBS and re-suspended in 1 mL PI staining solution (1 mg/mL sodium citrate, 50 μg/mL PI, 10 μg/mL RNase A, 0.5% Triton X-100 ). The cells were finally incubated at 37 °C for 30 min in dark. The distribution of cells in the cell cycle was measured by MultiCycle software of flow cytometry (Beckman Coulter, XL, Indianapolis, IN, USA).

### 4.7. Total Protein Extraction and Western Blot Assay

SGC-7901 cells were treated with 0.1625 μM, 0.325 μM, 0.65 μM *R*-PE for 24 h. For isolation of total protein fractions [36], cells were harvested and washed twice with PBS, and then the cells were lysed with cell lysis buffer (50 mM Tris-Cl, pH 8.0, 120 mM NaCl, 50 mM NaF, 200 μM sodium vanadate, 0.5% NP-40, 10 mM phenylmethylsulfonyl fluoride (PMSF) and 2 μg/mL aprotinin 0.2 μL, 10 μg/mL leupeptin 10 μL). The lysates were centrifuged at 12,000*× g* for 10 min at 4 °C, and the supernatant was saved at −20 °C. Protein concentrations of cell lysates were detected by Bradford assay [37]. Total protein samples were separated by polyacrylamide gelelectrophoresis (SDS-PAGE). The separated proteins were transferred to nitrocellulose filter membrane. After being blocked with blocking solution (5% skim milk in TBS, 10 mM Tris-HCl, 150 mM NaCl, pH 7.5 plus 0.1% Tween-20) at room temperature for 2 h, each membrane was incubated with primary antibodies overnight at 4 °C. Afterwards, the membranes were probed with the appropriate horseradish-peroxidase conjugated secondary antibody for 2 h at room temperature. Detection was performed by the 3,3*N*-diaminobenzidine tertrahydrochloride (DAB) Horseradish Peroxidase Color Development kit (Beyotime Institute of Biotechnology, Shanghai, China), according to the manufacturer’s instructions. Bands were recorded and relative density units of the bands were analyzed by a Gel Imaging System (Tanon, GIS-2019, Beijing, China). Densitometrical data of multiple experiments are shown. 

### 4.8. Statistical Analysis

The data is presented as the mean ± SD. Statistical significance was calculated using one-way ANOVA. In addition, *p*-values of ≤5% were considered to indicate statistically significant differences.

## 5. Conclusions

The current data demonstrates that *R*-PE induces apoptosis by arresting the SGC-7901 cell at S phase, which was achieved by decreasing the expression of the CDC25A protein, reducing the CDK2 protein activated and the formation of Cyclin-CDK complex.

## Figures and Tables

**Table 1:** Doses inducing 50% cell growth inhibition (IC_50_) of *R*-phycoerythrin (*R*-PE) against human gastric cancer SGC-7901 cells.

Groups	IC_50_ (μM)
*R*-PE	2.55
ADR	2.91

ADR, adriamycin.

**Table 2:** Apoptosis rate of SGC-7901 cells induced by *R*-PE.

Group	Concentration (μM)	Apoptosis Rate (%)
Control	—	4.03 ± 2.64
ADR	7.36	23.83 ± 2.02 ^b^
*R*-PE	1.30	12.37 ± 4.62 ^b^
	2.60	18.67 ± 3.06 ^b^
	5.20	24.50 ± 1.64 ^b^

^a^ *p* < 0.05; ^b^ *p* < 0.01 vs. control.

**Table 3:** The effects of *R*-PE on cycle phase of SGC-7901 cells.

Group	Concentration (μM)	G_0_/G_1_ Phase (%)	S Phase (%)	G_2_/M Phase (%)
Control	—	55.17 ± 5.96	23.17 ± 3.50	21.67 ± 6.01
ADR	1.84	50.93 ± 6.33	44.10 ± 0.70 ^b^	5.00 ± 6.46
*R*-PE	0.1625	67.97 ± 4.31	28.60 ± 1.66 ^a^	3.43 ± 5.95
	0.325	63.00 ± 8.43	33.43 ± 2.95 ^b^	3.57 ± 6.18
	0.65	56.17 ± 5.35	40.50 ± 2.26 ^b^	3.33 ± 3.09

^a^ *p* < 0.05; ^b^ *p* < 0.01 vs. control.

## References

[1] Zhao M.R., Sun L., Sun S.C., Gong X.Q., Fu X.J., Chen M. (2013). The 42.1 and 53.7 kDa bands in SDS-PAGE of *R*-phycoerythrin from Polysiphonia urceolata. Int. J. Biol. Macromol..

[2] Manirafasha E., Ndikubwimana T., Zeng X.H., Lu Y.H., Jing K.J. (2016). Phycobiliprotein: Potential microalgae derived pharmaceutical and biological reagent. Biochem. Eng. J..

[3] Galland-Irmouli A.V., Pons L., Lucon M., Villaume C., Mrabet N.T., Gueant J.L., Fleurence J. (2000). One-step purification of *R*-phycoerythrin from the red macroalga *Palmaria palmata* using preparative polyacrylamide gel electrophoresis. J. Chromatogr. B Biomed. Sci. Appl..

[4] Stadnichuk I.N. (1995). Phycobiliproteins-determination of chromophore composition and content. Phytochem. Anal..

[5] Wang L., Wang S.M., Fu X.J., Sun L. (2015). Characteristics of an *R*-phycoerythrin with two gamma subunits prepared from red macroalga *Polysiphonia urceolata*. PLoS ONE.

[6] Wilson K.M., Morrison I.E.G., Smith P.R., Fernandez N., Cherry R.J. (1996). Single particle tracking of cell-surface HLA-DR molecules using *R*-phycoerythrin labeled monoclonal antibodies and fluorescence digital imaging. J. Cell Sci..

[7] Bradford J.A., Buller G., Suter M., Ignatius M., Beechem J.M. (2004). Fluorescence-intensity multiplexing: Simultaneous seven-marker, two-color immunophenotyping using flow cytometry. Cytometry A.

[8] Selva C., Malferrari M., Ballardini R., Ventola A., Francia F., Venturoli G. (2013). Trehalose preserves the integrity of lyophilized phycoerythrin-antihuman CD8 antibody conjugates and enhances their thermal stability in flow cytometric assays. J. Pharm. Sci..

[9] Kim M.S., Kim T.S. (2013). *R*-phycoerythrin-conjugated antibodies are inappropriate for intracellular staining of murine plasma cells. Cytometry A.

[10] Hilditch C.M., Balding P., Jenkins R., Smith A.J., Rogers L.J. (1991). *R*-Phycoerythrin from the macroalga corallina-officinalis (rhodophyceae) and application of a derived phycofluor probe for detecting sugar-binding sites on cell-membranes. J. Appl. Phycol..

[11] Zhang F., Li J.F., Zou M.Q., Chen Y., Wang Y.F., Qi X.H. (2013). Simultaneous Detection of *Clavibacter michiganensis* subsp nebraskensis and *Pantoea stewartii* subsp. stewartii based on microsphere immunoreaction. J. Biomol. Screen..

[12] Munier M., Jubeau S., Wijaya A., Morancais M., Dumay J., Marchal L., Jaouen P., Fleurence J. (2014). Physicochemical factors affecting the stability of two pigments: *R*-Phycoerythrin of Grateloupia turuturu and B-phycoerythrin of Porphyridium cruentum. Food Chem..

[13] Huang B., Wang G.C., Zeng C.K., Li Z.G. (2002). The experimental research of R-phycoerythrin subunits on cancer treatment: A new photosensitizer in PDT. Cancer Biother. Radiopharm..

[14] Wu Q., Fu X.P., Sun L.C., Zhang Q., Liu G.M., Cao M.J., Cai Q.F. (2015). Effects of physicochemical factors and in vitro gastrointestinal digestion on antioxidant activity of *R*-phycoerythrin from red algae Bangia fusco-purpurea. Int. J. Food Sci. Technol..

[15] Senthilkumar N., Kurinjimalar C., Thangam R., Suresh V., Kavitha G., Gunasekaran P., Rengasamy R. (2013). Further studies and biological activities of macromolecular protein *R*-phycoerythrin from Portieria homemannii. Int. J.Biol. Macromol..

[16] Pan Q.W., Chen M.Z., Li J., Wu Y., Zhen C., Liang B. (2013). Antitumor function and mechanism of phycoerythrin from Porphyra haitanensis. Biol. Res..

[17] Sonani R.R., Singh N.K., Awasthi A., Prasad B., Kumar J., Madamwar D. (2014). Phycoerythrin extends life span and health span of *Caenorhabditis elegans*. Age.

[18] Daniel B., DeCoster M.A. (2004). Quantification of sPLA2-induced early and late apoptosis changes in neuronal cell cultures using combined TUNEL and DAPI staining. Brain Res..

[19] Marcellus R.C., Lavoie J.N., Boivin D., Shore G.C., Ketner G., Branton P.E. (1998). The early region 4 orf4 protein of human adenovirus type 5 induces p53-independent cell death by apoptosis. J. Virol..

[20] Jia S.G., Wang X.M., Qian H., Li T.Y., Sun J., Wang L., Yu J., Li X.G., Yin J.L., Liu T. (2014). Phylogenomic analysis of transcriptomic sequences of mitochondria and chloroplasts for marine red algae (Rhodophyta) in China. Acta Oceanol. Sin..

[21] Thomas D. (2002). Seaweeds.

[22] Sukhoverkhov S.V., Kadnikova I.A., Podkorytova A.V. (2000). Preparation of agar and agarose from the red algae Ahnfeltia tobuchiensis. Prikl. Biokhim. Mikrobiol..

[23] Ermak I.M., Reunov A.V., Lapshina L.A., Byankina A.O., Bratskaya S.Y., Sokolova E.V. (2013). Physicochemical and electron-microscopic study of carrageenans, sulfated polysaccharides from red algae of the families Tichocarpaceae and Gigartinaceae. Chem. Nat. Compd..

[24] Murugan K., Iyer V.V. (2013). Differential growth inhibition of cancer cell lines and antioxidant activity of extracts of red, brown, and green marine algae. In Vitro Cell. Dev. Biol. Anim..

[25] Zandi K., Tajbakhsh S., Nabipour I., Rastian Z., Yousefi F., Sharafian S., Sartavi K. (2010). In vitro antitumor activity of *Gracilaria corticata* (a red alga) against Jurkat and molt-4 human cancer cell lines. Afr. J. Biotechnol..

[26] Allmendinger A., Spavieri J., Kaiser M., Casey R., Hingley-Wilson S., Lalvani A., Guiry M., Blunden G., Tasdemir D. (2010). Antiprotozoal, antimycobacterial and cytotoxic potential of twenty-three british and irish red algae. Phytother. Res..

[27] Raman M., Doble M. (2015). kappa-Carrageenan from marine red algae, Kappaphycus alvarezii—A functional food to prevent colon carcinogenesis. J. Funct. Foods.

[28] Wang X.M., Zhang Z.S. (2014). The antitumor activity of a red alga polysaccharide complexes carrying 5-fluorouracil. Int. J. Biol. Macromol..

[29] Kazlowska K., Lin H.T.V., Chang S.H., Tsai G.J. (2013). In Vitro and In Vivo Anticancer Effects of Sterol Fraction from Red Algae Porphyra dentata. Evid. Based Complement. Altern. Med..

[30] Lin A.S., Engel S., Smith B.A., Fairchild C.R., Aalbersberg W., Hay M.E., Kubanek J. (2010). Structure and biological evaluation of novel cytotoxic sterol glycosides from the marine red alga *Peyssonnelia* sp. Bioorg. Med. Chem..

[31] George Rosenker K.M., Paquette W.D., Johnston P.A., Sharlow E.R., Vogt A., Bakan A., Lazo J.S., Wipf P. (2015). Synthesis and biological evaluation of 3-aminoisoquinolin-1 (2*H*)-one based inhibitors of the dual-specificity phosphatase Cdc25B. Bioorg. Med. Chem..

[32] Tilaoui M., Mouse H.A., Jaafari A., Zyad A. (2014). Differential effect of artemisinin against cancer cell lines. Nat. Prod. Bioprospect..

[33] Gao S.Y., Li J., Wang L., Sun Q.J., Gong Y.F., Gang J., Su Y.J., Ji Y.B. (2014). Ethanol but not aqueous extracts of tubers of* Sauromatum giganteum* (Engl.) Cusimano and Hett inhibit cancer cell proliferation. Asian Pac. J. Cancer Prev..

[34] Gao S., Tan H., Zhu N., Gao H., Lv C., Gang J., Ji Y. (2016). Oridonin induces apoptosis through the mitochondrial pathway in human gastric cancer SGC-7901 cells. Int. J. Oncol..

[35] Gao S.Y., Gong Y.F., Sun Q.J., Bai J., Wang L., Fan Z.Q., Sun Y., Su Y.J., Gang J., Ji Y.B. (2015). Screening antitumor bioactive fraction from *Sauromatum giganteum* (Engl.) Cusimano & Hett and sensitive cell lines with the serum pharmacology method and identification by UPLC-TOF-MS. Molecules.

[36] Gao S.Y., Li J., Qu X.Y., Zhu N., Ji Y.B. (2014). Downregulation of Cdk1 and CyclinB1 Expression Contributes to Oridonin-induced Cell Cycle Arrest at G (2)/M Phase and Growth Inhibition in SGC-7901 Gastric Cancer Cells. Asian Pac. J. Cancer Prev..

[37] Bradford M.M. (1976). A rapid and sensitive method for the quantitation of microgram quantities of protein utilizing the principle of protein-dye binding. Anal. Biochem..

